# A qualitative study on community perceptions on quality of healthcare services they received in the Malaria Elimination Demonstration Project in district Mandla, India

**DOI:** 10.1186/s12936-022-04400-6

**Published:** 2022-12-03

**Authors:** Mrigendra P. Singh, Harsh Rajvanshi, Praveen K. Bharti, Aparup Das, Vikesh Thakre, Himanshu Jayswar, Ram Shankar Sahu, Vinay K. Telasey, Altaf A. Lal

**Affiliations:** 1 Malaria Elimination Demonstration Project, Mandla, Madhya Pradesh India; 2 Indian Council of Medical Research – National Institute of Research in Tribal Health (ICMR-NIRTH), Jabalpur, Madhya Pradesh India; 3 Directorate General of Health Services, Government of Madhya Pradesh, Bhopal, Madhya Pradesh India; 4 Department of Health Services, Government of Madhya Pradesh, Mandla, Madhya Pradesh India; 5 Foundation for Disease Elimination and Control of India (FDEC India), Mumbai, Maharashtra India; 6 Present Address: Asia Pacific Leaders Malaria Alliance (APLMA), Singapore, Singapore; 7 Present Address: Indian Council of Medical Research – National Institute of Malaria Research (ICMR-NIMR), New Delhi, India

**Keywords:** Community perception, Quality healthcare services, Optimal utilization, Treatment-seeking behaviour, Malaria elimination

## Abstract

**Background:**

The utilization and impact of the healthcare services depend on the perceived quality, appropriateness, ease of availability, and cost of the services. This study aimed to understand the community's perception of the quality of healthcare services delivered as part of the Malaria Elimination Demonstration Project (MEDP), Mandla, Madhya Pradesh, India.

**Methods:**

The study used qualitative techniques to analyze the community perceptions that emerged from the participants’ narratives during the Focus Group Discussions (FGDs) and in-depth Interviews with Key Informants (IKIs) on the promptness and quality of healthcare service delivery, the behaviour of MEDP staff, Information, Education and Communication, and Behavioural Change Communication activities, coordination with community members and other health personnel, and capacity building of healthcare workers and the community.

**Results:**

36 FGDs and 63 IKIs with 419 respondents were conducted in nine blocks of district Mandla. Overall, 97% to 100% of beneficiaries associated MEDP with regularity and prompt service delivery, availability of diagnostics and drugs, friendly behaviour, good coordination, and community mobilization to enhance treatment-seeking behaviour.

**Conclusions:**

The study's findings highlighted the importance of building and maintaining the community's participation and promoting the demand for optimal utilization of healthcare services inside the village to promptly achieve the malaria elimination goal.

## Background

Malaria continues to be a significant public health problem, with an estimated 241 million malaria cases and 0.627 million malaria-attributed deaths globally in 2020 [1]. The World Health Organization (WHO) South-East Asia region accounted for five million malaria cases, contributing 2% of the global estimated malaria burden in 2020. India accounted for 83% of the cases in the region [1]. Due to the disruption of the anti-malarial services during the COVID-19 pandemic, about a 6% increase in malaria cases was recorded in 2020 compared to 2019 [1].

Most of the rise during this period was reported in the WHO African region. Between 2019 and 2020, malaria cases increased in all 11 high-burden and high-impact countries except India due to disruption to access to malaria diagnosis and treatment. The National Centre for Vector Borne Disease Control (NCVBDC), the Ministry of Health and Family Welfare, India, reported a 46% decrease in malaria cases in 2020 compared to 2019. This achievement was attributed to uninterrupted community-based fever surveillance mainly provided by the frontline community healthcare workers, such as the Accredited Social Health Activists (ASHAs). ASHAs were also recognized at the 75th World Health Assembly (WHA) for the crucial role they have played in “linking the community with the health system, to ensure those living in rural poverty can access primary health care services, as shown throughout the COVID-19 pandemic” [2].

Madhya Pradesh (MP) was among the most malarious states in India contributing 8% of the total reported malaria cases and 7% deaths during 2012 to 2016. *Plasmodium falciparum* and *Plasmodium vivax* are the two dominant species with seasonal variation and *Anopheles culicifacies* is the main malaria vector. District Mandla is one of the most malarious and a tribal dominant district in the state [3].

During the period from April 2017 to March 2021, a model project known as the Malaria Elimination Demonstration Project (MEDP) was conducted in district Mandla of MP, India, to demonstrate that eliminating malaria was feasible in all 1233 villages of the district of 1.15 million population. The lessons learnt from MEDP were envisaged to help eliminate malaria from the rest of the state and the country. MEDP was a first-of-its-kind public–private-partnership between the Indian Council of Medical Research (ICMR) through the National Institute of Research in Tribal Health (NIRTH), the Government of Madhya Pradesh (GoMP), and the Foundation for Disease Elimination and Control of India (FDEC-India, established by Sun Pharmaceutical Industries Ltd. as a not-for-profit entity).

The MEDP used the T4 (Track fever, Test fever, Treat patient, and Track patient) strategy for robust active surveillance along with monitoring of vector control interventions, such as the Indoor Residual Spraying (IRS) and the distribution of Long-Lasting Insecticidal Nets (LLINs), which have been published as thematic series [4–7].

The project also used Information Education Communication and Behavior Change Communication (IEC and BCC) strategies and robust monitoring and evaluation of all components of the project [5, 6]. At the community level, a total of 235 trained Village Malaria Workers (VMWs) and 25 Malaria Field Coordinators (MFCs) worked to deliver healthcare services at the doorstep [5]. The project achieved a 91% reduction in indigenous malaria cases in the district [7], and zero indigenous cases were reported for four consecutive months from December 2020 to March 2021 [8].

The effectiveness of any healthcare intervention programme depends on the optimal acceptance and utilization of the services delivered in the community. The utilization of healthcare services by an individual is associated with the perceived quality and appropriateness of the services offered, their easy availability, cost of healthcare, and socio-economic status of the individual, including income, the standard of living, and trust in the local traditional medicine [9–12]. The perceived healthcare quality affects consumer decisions about whether and where to seek health care [13, 14]; hence, it is essential to understand the factors affecting community behaviour toward the uptake of healthcare services [12].

Therefore, this study was conducted to understand the perception of the community, beneficiaries and stakeholders regarding the services delivered by the staff of MEDP in district Mandla. Critical assessments included the promptness and quality of healthcare service delivery, IEC and BCC activities, coordination with community members and other health personnel, and capacity building of community healthcare workers and the community.

## Methods

### Study sites and population

District Mandla located between coordinates of 22° 02′ and 23° 22′ N latitudes and 80° 18′ and 81° 50′ E longitudes in the east-central region of the state of MP, India. Total population of the district was 1.055 million, sex ratio was 50.21% females and 49.79% males and total literacy was 57% as per census 2011. About 58% population were belonging to the ethnic tribal groups mainly “*Gond*” and “*Baiga*” [15]. The study was conducted in the nine administrative blocks of district Mandla, MP, India, and categorized into villages, blocks, and the district. Respondents for the present study included the following: (1) at the village level: frontline community healthcare workers known as the ASHAs, the locally elected community leader called as ‘*Sarpanch*’, and male and female community members; (2) at the Block level: Block Medical Officers (BMO), Block Programme Managers (BPM), Malaria Inspectors (MI) and Malaria Technical Supervisors (MTS), Auxiliary Nurse Midwives (ANMs), high school teachers, and high-school students; and (3) at the District level: Officers of forest, agriculture and revenue department (inter-sectorial stakeholders). The health officers from the government of MP and FDEC-India at the district level were excluded from the survey because of a potential conflict of interest as they were part of implementing the project. All the study participants were representative of a mixed population of the “Scheduled Castes (SC)”, “Scheduled Tribes (ST)”, “Other Backward Castes (OBC)” and “General Castes” categories and both sexes—males and females.

### Procedures

A team of three interviewers with undergraduate and postgraduate qualifications and minimum 5 years of experience in conducted community health surveys were assigned. Before data collection, a one-week training workshop was conducted for the interviewers. The workshop focused on the basics of malaria elimination, qualitative research methods, and research ethics. The questionnaire guide was developed in English and local languages *(Hindi)*. A pilot survey was conducted to validate the survey tools and to sensitize the team on the study procedures and complexities that might arise during data collection.

### Sampling method

The present study used Focus Group Discussions (FGDs) and in-depth Interviews with Key Informants (IKIs). The purposive sampling method was adapted. ASHAs from randomly selected ten villages in each block were invited to a central location/health sub-centre as per their convenience for the FGD. The FGD with a group of ten male and ten female community members were conducted separately at community hall or “Panchayat building” of any one of the randomly selected village in each block. The adjoining village was considered for data collection if the study participant of the selected village was not available to participate in the study at the time of visit.

At the block level, IKIs were conducted for BMOs, BPMs, MIs or MTS, ANMs, and high school teachers and *“Sarpanch”*—is a locally elected community leader at the village level, who is responsible for the development and day-to-day functioning of the village. At the same time, FGDs of the students in high school were also conducted at each block. Furthermore, IKIs were conducted for the officers from the agriculture, forest, and revenue departments at the district level.

Interviewers carried out the FGDs as a three-personnel team. One team member conducted the FGD; the second member was a moderator and worked to ensure the active participation of each study participant. The third member took notes and was responsible for proper recording using a voice recorder. The discussions/interviews were conducted in the local language *(Hindi)* using a semi-structured questionnaire guide. The guide covered specific topics and enabled open-ended exchanges and probing. The interviewers started discussion on some light topic of local interest with the group of participants for ice breaking and built rapport. Each FGD/IKI took 60–90 min to complete. Each of the study participants who participated in FGD/IKI was provided a workshop bag containing a notepad and pen. Before conducting the discussion/interview, the aims and objective of the study were explained. Verbal consent was taken from all the adult study participants. Support from the headmaster of the visited high school was sought before conducting a study in the school. Verbal assent from the high school students was obtained in the presence of their class teachers as their legally authorized representative. None of the study participants declined participation in the study.

### Data analysis

The notes of the note-taker were validated with the corresponding voice recordings. These notes and the embedded quotations were transcribed into English, and initial thoughts were listed. The transcribed data was further verified with the voice recording to ensure the accuracy of the transcription. The multiple readings of the transcripts and listening to voice recordings helped in the complete immersion of the investigators to achieve familiarity with the qualitative data [16]. Further, the transcribed notes were coded using the grounded theory [17]. The interesting instances which emerged from the group discussions and interviews were coded and further grouped into categories which had similar concepts. The themes derived from the data analysis were listed and recorded in Microsoft Excel 2013. These identified themes were used to validate the findings and reduce the bias using data triangulation from different categories/sources of study participants. The responses were analysed by stratification of the community health workers, health officers/programme managers, community members, and other inter-sectorial stakeholders. The COREQ: 32 items checklist of the reporting qualitative studies has been followed [18].

## Results

### Background characteristics of the respondents

The present study comprised 36 FGDs and 63 IKIs, totaling 419 respondents in nine blocks of the Mandla district. Each FGD consisted of 8 to 11 respondents. The gender distribution of the respondents was 57.52% females and 42.28% males. More than half of the study participants (53.22%, 223/419) belonged to the “Scheduled Tribes”, followed by 36.04% (151/419) “Other Backward Castes”, 8.35% (35/419) “Schedule Castes” and 2.39% (10/419) “General Castes” groups. The median age of the respondents was 38 (ranging between 12 and 72) years. The educational status of village-level frontline healthcare workers (ASHAs) was class 8th to graduate, and the community members had completed 5 to 12 years of schooling. *Sarpanches* were educated from class 10th to graduation; other vital informants were higher-level graduates (Table 1).

**Table 1:** Demographic information of the study participant

Participants	Sex		Caste category				Age (years)			Education	
	M	F	ST	SC	OBC	GEN	Mean	Min.	Max.	Min.	Max.
FGD											
ASHAs^┼^		91	53	12	25	1	37.73	26	55	8	Graduate
Male^*^	90		54	6	24	6	44.64	24	68	5	12
Female^*^		95	45	5	42	3	40.70	21	72	0	10
Student	40	40	35	1	44	0	13.82	12	16	7	8
In-depth Interview											
BMOs	8	1	5	3	1	0	41.32	39	55	MBBS	MD
MIs	7	2	4	3	2	0	46.53	41	53	Graduate	
BPM	8	0	3	0	5	0	34.21	38	45	Graduate	MBA
ANMs	0	9	7	0	2	0	39.54	29	51	12 + ANM	Graduate
Sarpanch^*^	8	1	6	0	3	0	37.61	32	55	10	Graduate
Teacher	7	2	4	3	2	0	39.52	34	59	Graduate	Post Graduate
Forest Officer	4	0	3	1	0	0	33.09	30	39		
Revenue Officer	3	0	2	1	0	0	31.67	28	35		
Agriculture Officer	3	0	2	0	1	0	36.67	31	42	Graduate	Post Graduate

^┼^Main occupation of ASHAs was housewives; ^*^Main occupation of male and family community members and Sarpanch was casual labourers and agricultural activities on own land

The thematic analysis of the respondent’s perception regarding the MEDP work for malaria elimination in district Mandla was presented below by stratifying the healthcare providers, program managers, community members, and other inter-sectorial stakeholders (Table 2).

**Table 2:** Illustrative statements of the study participants during FGDs and IKIs

Participants	Themes		
	Work proficiency, behavior and connecting to the beneficiaries	Coordination with stakeholders	IEC/BCC and capacity building
ASHAs	“During MEDP period, people called MEDP workers when they felt sick. However, we had never informed so that we lost our incentives and discouraged” “These workers were most popular in the community due to their prompt availability and better quality of service delivery. Most of the time community preferred to contact MEDP workers for seeking treatment of fever which caused losses of incentive paid to us for our services towards malaria diagnosis and treatment”	“ASHAs are working for various health program delivery, and have overburden. MEDP workers helped in diagnosis and treatment of fever cases and pulse, polio, covid vaccination program. MEDP workers also helped in distribution of LLIN and monitoring of regular use of bednet and IRS work”	“MEDP has been provided printed literature in local language ‘Hindi’ and images related to malaria etiology, preventive measures, diagnostic methods, treatment dose chart according to the malaria species. MEDP also provided intensive training on malaria diagnosis and treatment to the ASHAs of the district”
Community members			
Female	“During MEDP period we got the treatment at our door step. Now when MEDP was closed we didn't get the facility inside the village and visiting Mandla which is about 35 km away as we have no other option and it not only expensive but also time consuming. Most of the time two or three visits are required for getting complete treatment” “They always asked for our wellness even when they found us on the way. They are very much polite and respect to all the elders of the village”		“MEDP workers motivated the community for regular use of bednet, aware to clean surrounding, remove water to avoid mosquito breeding. Keep clean and covered the drinking water and change it regularly. I feel that now we felt sick very rarely particularly regarding episodes of febrile illness was very rare in the community”
Male	“Earlier we spent money to seek treatment, went district government or private hospitals which was expensive and time consuming. These all effects badly to our economic condition mainly during crop season we lost lot of money and time. MEDP workers helped us a lot and provide prompt treatment at our door step” “Few years ago, when MEDP not existed, almost every family suffered from fever with chills and rigor due to malaria. Now there is no malaria case in our knowledge” “MEDP workers provided treatment only for malaria. This type of facility should be provided for all the common diseases in villages”		“MEDP staff repeatedly provided knowledge on mosquito and malaria, therefore, our community is now considerably awaked and taking care for their health and hygiene. The villagers now seek treatment as early as possible from ASHA or ANM or visiting PHC/CHC”
Sarpanch (elected community leader)	“Few years ago, almost every family experienced from malarial fever in the villages particularly during rainy season. Now we didn't see any malaria cases in our area” “MEDP staff were worked very sincerely. Frequently visited each and every house to provide treatment of fever cases. They shared mobile number to contact them any time if we felt sick”	MEDP staff and ASHA, ICDS worker of the village, all together, occasionally visited households in the village	MEDP staff regularly organized health education campaign programme in the villages, weekly haat market, school and panchayat to motivate the villagers against malaria and other common health issues. They used various methods for awareness such as poster, slogan and song in local language, rally, road show etc
Block level healthcare providers*	“Malaria cases in district was already controlled before the MEDP and very less number of malaria cases was reported. However, recently almost zero cases reported in the district” “MEDP contributed remarkable work for malaria elimination in the area. Staff were very sincere, hardworking and friendly. They test, treat and track each malaria cases, monitored all travelers to check the imported malaria cases in the area. I strongly believe that this type of intensified work is essentially needed for malaria elimination” “The proper management and regular monitoring of the field activity in MEDP project was good” “MEDP program objective was good and their work was also appreciable but the time when project was introduced was not correct as malaria situation was already under controlled. I think this was not required at that time in the district and it was total wastage of resources” “Each MEDP village workers have to work in 4 to 5 villages and due to distance between villages usually their visit in village was delayed as when they reach village the villagers moved out from house for their livelihood and not able to contact. Also some of the areas were mostly ignored due to difficult terrain and hard to reach”	“MEDP field staff were work sincerely in coordination with state health staff and with their joint efforts we achieved the malaria elimination goal in the area.” “Each positive cases were reported and shared with the group of state program personals including me to regular track the malaria cases which help in malaria control in the area” “During the strike of state community health worker, MEDP staff help us to deliver health services in villages particularly in pulse polio vaccination programme”	“Mandla district is a tribal dominated district and rural community are less aware and poor health seeking practices which required the continuous efforts to educate and motivate for enhancing the health awareness. MEDP contributed very much in this regard” “MEDP conducted IEC/BCC campaign in weekly market to aware community against malaria. Motivated community for regular use of bednet, aware to clean surrounding, remove water to avoid mosquito breeding. Also conducted meeting at village level and in school to aware the community against malaria” “ASHAs and ANM are trained by state health program and time to time they get training at CHC during monthly meeting. However, MEDP also provided intensified training to ASHAs and ANM towards malaria diagnosis and treatment during the project period”
High school teachers	“MEDP staff behave very well and mixed with the community like a family member. They worked very sincerely for awareness enhancement towards malaria and other mosquito borne disease, symptoms, prevention, diagnosis and treatment. Students sprayed this information in their family and help in community awareness”	“Visits of MEDP staff in school was unscheduled and they visited without any pre-intimation which created inconvenience and disturbing to the scheduled teaching work. Occasionally, it also created conflict and unhealthy relation”	“MEDP staff were explained very well to children and also teachers regarding malaria disease, source of infection, preventive measures, symptoms, diagnosis and treatment. As a result of frequent awareness program people were using bednets regularly and very particular to use bednet during rainy season. Therefore malaria cases was controlled in our area”
District level other stakeholder**	“During the MEDP, all fever cases attended and treated at door step promptly by the MEDP staff, they were available at the village itself. But now we are visiting CHC for getting treatment when felt sick”		“They provided their services to aware against malaria to our ground level staff. They provided diagnosis and treatment for malaria on spot and immediately when we required their services” “MEDP staff organized regular health awareness campaign to motivate the community and awareness enhancement towards malaria”

^*^Block level healthcare providers like BMO, BPM, MI, MTS, ANM; **Forest, revenue, agriculture officers at district level

### Work proficiency, behaviour, and social connection to the beneficiaries

Almost all (97%, 406/419) respondents reported that MEDP staff regularly performed active door-to-door surveillance, diagnosis, and treatment and tracked each malaria-positive case (*T4 strategy: Track fever, Test fever, Treat patient, Track patient).* All (100%) community FGD respondents informed that the village-level staff shared their contact numbers with the household and wrote them at the doorframe whenever their services were required outside the regular visits.

One female community respondent stated, "*During the MEDP period, we got the treatment at our doorstep. Now when MEDP has gone, we do not get the facility inside the village and are forced to visit Mandla, which is about 35 km away, as we have no other option, and it is not only expensive but time-consuming. Most of the time, two or three visits are required for complete treatment”.* An ASHA respondent of one FGD stated*, "These workers were most popular in the community due to their prompt availability and better quality of service delivery. Most of the time, the community preferred to contact MEDP workers to seek treatment of fever which caused losses of incentive paid to us for our services towards malaria diagnosis and treatment”.* Another ASHA responded*, "We are working for various health programmes including malaria on an incentive basis. While MEDP staff worked only for malaria diagnosis and treatment but were paid much higher than the ASHAs”.*

Most (99%, 415/419) of the respondents informed that the behaviour of ground-level MEDP staff was good and that they were well connected with the community. A female community respondent stated, “*They always asked about our wellness whenever they found us on the way. They were very polite and respectful to all the village elders”*. At the block level, health providers recognized the significant contribution of MEDP towards malaria elimination in the district.

### Coordination with stakeholders

Most (99%, 224/227) of the respondents informed that MEDP staff worked in good coordination with state front-line health providers such as ASHAs, ANMs and Integrated Child Development Scheme (ICDS) workers. An array of activities included delivery of healthcare services, distribution, and monitoring of LLIN, IRS work, health awareness programme, pulse-polio (a government-led vaccination drive to eliminate the polio from the community in India) and COVID-19 vaccination programme.

### Contributed towards community awareness, health-seeking behaviour, and capacity building

Most (99%, 416/419) of the respondents, such as the ASHAs, male and female community members, *sarpanch*, teachers and students, block level healthcare providers and other stakeholders firmly recognized that the MEDP staff worked sincerely to motivate the community for seeking early diagnosis and treatment. MEDP also organized periodic health camps in weekly markets (*Haat bazaars)*, village panchayats, and schools to mobilize the community for health awareness.

During an FGD, one ASHA stated, "*MEDP has provided us with printed literature in local language and images related to malaria aetiology, preventive measures, diagnostic methods, treatment dose chart according to the malaria species. MEDP also provided intensive training on malaria diagnosis and treatment to all the ASHAs of the district*”. A male community member stated that *“MEDP staff repeatedly provided knowledge about mosquito and malaria. Therefore, our community is now considerably awakened and is taking care of their health and hygiene. The villagers now seek treatment as early as possible from ASHA or ANM or visiting PHC/CHC for treatment”.* At the block level, a healthcare provider stated, "*District Mandla is a tribal dominated district. The rural communities are less aware and have poor health-seeking practices requiring continuous education and motivation efforts. MEDP contributed very much to this regard”.*

## Discussion

The Malaria Elimination Demonstration Project has meticulously documented its operational and technical findings and disseminated them to the scientific community, malaria elimination programmes, and policymakers [4–7, 19–26]. While the scientific rigour is noteworthy, it was essential to assess the project's performance from the end user's perspective, i.e., the beneficiaries (community) of MEDP. Hence, the present paper is a first-of-its-kind study in India that describes the qualitative assessment of the beneficiaries (community) and the collaborators at the district level.

In the present study, the ASHAs expressed their concern over the loss of incentives due to the preference of the community to seek malaria services from MEDP staff. Under the National Health Mission, ASHAs receive performance-based incentives to deliver first-contact healthcare services such as immunizations, reproductive and child health referrals, diagnosis and treatment of malaria cases in the community, etc. Every ASHA is envisaged to champion community participation in public health programmes in her village [27]. The incentives given to the ASHAs are INR 15 [0.19 USD (INR 1 = 0.013 USD)] for diagnosis of malaria and INR 75 (0.98 USD) for providing complete treatment of each malaria-positive case as per national and state guidelines [28]. In comparison, the Village Malaria Workers and Malaria Field Coordinators of MEDP Mandla were paid a flat rate salary, which included diagnosis and treatment of any number of malaria cases along with IEC/BCC, monitoring of vector control interventions, assisting the state in other health programmes (within a defined geographical area) etc. [5]. It is worth mentioning that MEDP focused on building the capacity of ASHAs by performing a malaria elimination needs assessment followed by comprehensive training to ensure smooth transitioning of responsibilities after the closure of MEDP field activities [24].

Historically, tribal areas have been associated with poor health-seeking behaviour due to the lack of awareness, sub-par healthcare services and a below-average rate of satisfaction in the community [29–31]. MEDP meticulously enhanced the community’s knowledge and understanding of malaria and other vector-borne diseases [5, 6, 24]. Most of the respondents recognized that there was a positive change in the health-seeking behaviour of the community. This change in community behaviour is notable because of the extensive training and capacity building at the grassroots level by MEDP, which needs to be sustained and adequately funded to realize the 2030 target of malaria elimination. One of the reasons behind the success of polio elimination was a dedicated cadre of grassroots professionals responsible for administering immunizations and conducting surveillance [32]. A similar workforce strategy would be desirable in which the village-based ASHAs, who are responsible for almost all health programmes, are joined by a dedicated staff focusing only on malaria elimination, especially in high-burden districts. The modified strategy that strengthens ASHA’s capacity would also be useful in conducting Mass Screening and Treatment (MSaT) if required for malaria elimination in high burden districts in India. This new approach would ensure that all fever cases that test positive for malaria get the proper treatment.

Alongside the IEC/BCC strategy of MEDP and the continuous follow-up of every malaria-positive case under the T4 strategy helped build faith in the community [7] is also desirable for frontline workers for malaria elimination to consider. The high levels of satisfaction with the services of MEDP reported in the present study can be attributed to strict adherence to an Advance Tour Plan by the VMWs and MFCs, regular availability of diagnostic and drugs, robust training, monitoring and accountability framework, representation from the local community, and sustainability principles of the project [5, 23, 26]. Similar findings were reported by other studies conducted in Odisha and Madhya Pradesh [30, 33, 34].

As part of the operational strategy, MEDP achieved its goal of indigenous malaria elimination and ceased the field operations by March 2021 in a phased manner. Following this, community-level healthcare workers like ASHAs, ANMs and MPWs performed the diagnosis and treatment of fever cases. The findings of this study highlighted the issues arising due to the non-availability of MEDP services in the Mandla district in terms of rising out-of-pocket expenditures (OOPE).

There is a multifold increase in the OOPE accompanied by loss of daily wages and illnesses during crop cultivation/harvesting seasons. India is already struggling with the increasing OOPE costs the commoner bears. The mean annual OOPE is reported to be 14,660 INR (186.4 USD) and 21,564 INR (274.3 USD) for kids less than 1-year-old, against net national income (NNI) of 135,000 INR (1687 USD). These costs are split into 43% for pharmacies, 28.5% for private general hospitals, 7.42% for public government hospitals, 6.8% for medical and diagnostics and 6.26% for providers of patient transportation and emergency rescue [35].

MEDP Mandla has not only eliminated the indigenous transmission of malaria but would have eliminated the malaria component of the OOPE during its operations in the district. The project has developed a sustainable model that can replicate similar results in the state and the country without the additional cost of the human resources [23].

This study has also identified the critical role of capacity building of providers of health care in disease control and elimination programs. In the context of malaria elimination, the malaria transmission can be interrupted by focusing on the Prescriber community (physicians that use guidance provided by the program), Provider workforce (ASHA/ANM/MPW that conduct surveillance, treat patients, and make tools of vector control available to residents in the people), and the People, who are beneficiaries at the community levels. In this network, the Providers are the critical link between the Prescribers and the People. Therefore, continued capacity building and their performance assessment should be conducted periodically for any additional training needs and capacity building needed for malaria elimination and eradication.

## Conclusion

The present study has highlighted the importance of community perceptions for an effective community-based healthcare service delivery. The efficient uptake of these services by the community is key to the sustainable transitioning of best health-seeking practices after the withdrawal of model demonstration projects. The study revealed that MEDP demonstrated success within a short span of time through key attributes such as willingness to collaborate with government health staff, regular surveillance, and quality of services delivered to the community. Lack of continuity between model interventions and the existing healthcare system exposes the community to the risk of a resurgence of malaria, which will have long-term debilitating effects on the health and economic state of the community.

## Limitations

The authors have undertaken several efforts to minimize any bias in the study. One of the limitations could be that the respondents knew that the study team was engaged in the MEDP which sometimes may affects the independent views of the respondents. While the moderator of the team ensured active participation of the FGD group, few respondents hesitated to speak in group.

## Data Availability

We have reported all the findings in this manuscript. A copy of the data (recorded voice) is stored with MEDP. If anyone wants to review or use the data, they should contact: Dr Altaf A. Lal Project Director—Malaria Elimination Demonstration Project, Mandla Foundation for Disease Elimination and Control of India, Mumbai, India 482003 E-mail: altaf.lal@sunpharma.com, altaf.lal@gmail.com.
